# Is neuregulin-1 (NRG-1) a potential blood biomarker linking depression to obesity? A case-control study

**DOI:** 10.1186/s12888-023-05160-6

**Published:** 2023-09-14

**Authors:** Heba Ahmed Abdelaziz, Tamer Nabil Abdelbaki, Yomna E. Dean, Sara Assem

**Affiliations:** 1 Family Health, Mental Heath Department, High Institute of Public Health, Alexandria, Egypt; 2 Faculty of Medicine, General Surgery Department, Alexandria University, Alexandria, Egypt; 3 Faculty of Medicine, Alexandria University, Alexandria, Egypt; 4 Faculty of Medicine, Medical Biochemistry Department, Alexandria University, Alexandria, Egypt

**Keywords:** Depression, Obesity, Neuregulin-1, Gastrectomy

## Abstract

**Background and aim:**

No definite biomarker linking depression and obesity has been found yet. Our study aimed to investigate neuregulin-1 (NRG-1) as a potential blood biomarker for this association.

**Methods:**

A case–control study was conducted on 108 obese subjects assigned for laparoscopic sleeve gastrectomy and 100 non-obese controls. Depression was assessed pre- and post-operatively. Serum NRG-1 was measured.

**Results:**

Pre-operatively depression was significantly higher among obese compared to non-obese patients. After the operation, 1.9% of the severely depressed subjects reported no depression, while 5.6% became moderately depressed; about 6% of the moderately depressed and 16% of the mildly depressed became not depressed. Serum NRG-1 level was significantly lower among obese and severely depressed compared to the controls.

It was negatively correlated to the level of depression pre- and post-operative (*r* = -0.764 and -0.467 respectively). The sensitivity of serum NRG1 as a predictor for depression pre- and post-operative was 92.45% and 52.94% respectively. Specificity was 69.09% and 79.73% respectively at cut-off values of ≤ 3.5 and ≤ 2.5 ng/ml.

**Conclusion:**

NRG-1 is a possible biomarker for the diagnosis of depression pre-bariatric surgery and the prediction of its prognosis post-operatively.

## Introduction

Both depression and obesity are highly prevalent as well as comorbid problems with major implications and a huge burden [1, 2].

Depression involves persistent sadness and loss of interest among other harsh symptoms interfering with normal life [3, 4]. It has several genetic and environmental contributions [5]. Globally, depression ranges from 2 and 6%, with women and older age being more affected [6], and people affected increase consistently [7]. In 2019, the prevalence of depression in Egypt was 4.13% [6].

Obesity, having tripled over the past four decades, further presents a risk to health and functioning [8–10]. The WHO estimates that 39% of the human adult population is overweight and 13% is obese [9, 11].

Growing evidence suggests complex two-way relationships and parallels growth between depression and obesity [12, 13]. Several common risk factors have been identified, including genetic factors and previous psychiatric disorders [2, 14, 15]. Recent research exposes the contribution of obesity to depression through neural circuits, biological mechanisms, low self-esteem, and stigmatization [5, 16–18]. The comorbidity increases the risk for chronic diseases such as cardiovascular disease, type 2 diabetes, and asthma [12, 15, 19, 20]. Depression is usually associated with weight gain and obesity, in turn, the incidence of depression and anxiety is amplified by obesity [13]. Many obese patients treated for depression respond poorly to therapy, suggesting that obesity may reduce the efficacy of antidepressant treatment [21].

Neuregulins (NRG) are polypeptide growth factors belonging to the epidermal growth factor family that signals through receptor tyrosine kinases encoded by the erythroblastic leukemia viral oncogene homolog receptor (ErbB) family [22, 23].

Neuregulin-1 (NRG-1), has been known as a stress-mediated transmembrane growth factor and an active component of the epidermal growth factor (EGF)-like family. Its function is vital in promoting the growth and development of the central nervous system (CNS), neural remyelination after injury, immunomodulatory responses, and synaptic plasticity [24–30]. Hence, NRG-1 deficiency within the cortical projection neurons results in increased inhibitory connections [31, 32], which is consequently associated with chronic stress and depression [23]. Interestingly, NRG-1 treatment increases serum leptin sensitivity and improves insulin sensitivity, in obese mice [28, 33–35].

For morbidly obese patients, bariatric surgery is one of the most effective treatments. Laparoscopic sleeve gastrectomy (LSG) has become one of the most performed bariatric operations as it is associated with successful short-term weight reduction and a positive impact on different comorbidities [36–38]. Moreover, it leads to remission in obesity-related diseases including type 2 diabetes, non-alcoholic fatty liver, and obstructive sleep apnea. The most common complications of sleeve gastrectomy are bleeding, severe vomiting, nutrient deficiencies, and leakage [38].

Nonetheless, research to explore the sophisticated relation of sleeve gastrectomy to different mental and psychological disorders has been highlighted with controversial results [39, 40]. In this context, the present study is a trial to identify whether NRG-1 can be a blood biomarker that relates obesity to depression and its effect on post-LSG weight reduction and improvement of depression symptoms.

## Aim of the study

### General objective

The present work aims to investigate the role of neuregulin-1 (NRG-1) as a potential blood biomarker linking depression to obesity among obese subjects assigned for laparoscopic sleeve gastrectomy, attending Alexandria Main University.

### Specific objectives

1. To assess depression symptoms among obese subjects assigned for LSG, attending Alexandria Main University.
2. To measure NRG-1 serum level as a maker for depression among obese subjects.
3. To investigate whether NRG-1 serum level could be used as a predictive marker for depression among obese subjects.
4. To assess the effect of LSG on depressive symptoms among obese subjects.

## Plan of the study

### Study setting

This study was conducted at Alexandria Main University.

### Study design

A case–control study was conducted.

### Target population

The study population included obese patients attending the above-mentioned setting, and normal-weight subjects as a control group.

#### Inclusion criteria

Any obese person scheduled for LSG in the above-mentioned setting was asked to join the study.

#### Exclusion criteria

Those who were already on antidepressants or anxiolytic medication or psychotherapy for depression or anxiety were excluded from the study. Any participant who was on any weight reducing medicine, supplement, or even special diet within 1 year before surgery was excluded from the study.

### Sample size

As the prevalence of depression among obese adults was estimated to be 23% [41], a minimum sample size of 100 subjects was required to achieve a minimum power of 80% for detecting a change in the percentage value of sensitivity of NRG1 to detect depression among obese adults from 0.50 to 0.80, based on a target significance level of 0.05. The sample size was calculated by using PASS software. Another 100 subjects were required as controls, making the total sample size 200 subjects.

### Type of sample and method of selection

Cases and controls were assigned based on BMI, with obese patients having a BMI of 30 or above as cases and those having a BMI less than 30 as controls. The body mass index (BMI) is calculated by dividing the body weight in kilograms by the square of height in meters. For adults, current guidelines from the WHO define a normal BMI range as 18.5–24.9, ≥ 25 kg/m2 as overweight, and ≥ 30 kg/m2 as obese, with severe or morbid obesity defined as a BMI ≥ 40 kg/m^2^ [8–10].

### Data collection method

**Fig. 1:**
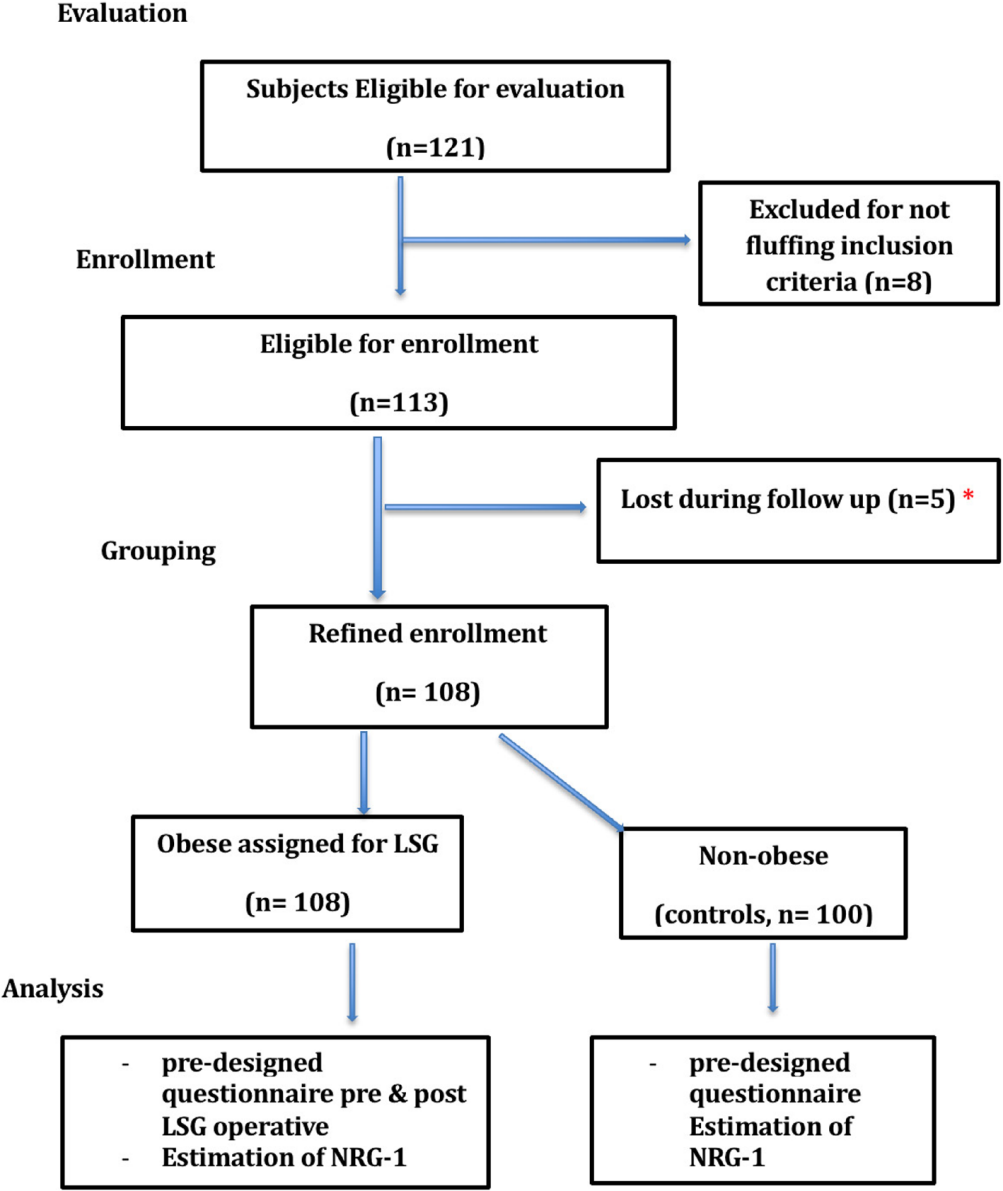
Flow diagram of the study population. * The cases who were lost during follow-up either developed postoperative complications or could not be reached to complete the second questionnaire

Participants were subjected to an assessment questionnaire to confirm eligibility as well as depression symptoms and NRG-1 level. After the target number of attendants was reached, a close number was set for control. The laparoscopic sleeve gastrectomy (LSG) bariatric operation was performed, then followed by the post-operative assessment 1 month after the operation. By the end of the study, participants were 108 subjects and 100 controls (Fig. 1). The cases who were lost during follow-up either developed postoperative complications or could not be reached to complete the second questionnaire.

#### Pre-operative phase (I) 

##### Structured interview using a pre-designed questionnaire to collect the following data

The following techniques and tools were used to identify subjects fulfilling the eligibility criteria:

- Socio-demographic data include age, gender, residence, marital status, duration of marriage, and number of children. (Questions (q.) 1–11)
- Personal data; history of chronic illness, regularly received medications. (q.12–15)
- Psychiatric history of women including; the presence of psychiatric illnesses, previous admission to a mental hospital, received medications or psychosocial interventions. (q16-20)
- Personal measurements; Weight, height, NRG-1. (q.21–23)

##### Arabic Version of Beck Depression Inventory (BDI-II) to assess depression symptoms [42, 43] (q.24–44)

BDI-II is a 21-item scale corresponding to DSM-IV criteria for diagnosing depressive disorders. The score is added up for each of the twenty one questions. Each question is scored from 0–3. The total score is categorized into; 0–13 for having no depression, 14–19 for mild depression, 20–28 for moderate, and 29–63 for severe depression [42, 43].

##### Blood sampling and NRG-1 serum level measurement

Fasting blood samples (3 ml) were taken from all the study participants. The samples were transferred on dry ice to the lab within less than 2 h. allowed to clot at room temperature, centrifuged then serum was separated and aliquoted in Eppendorf tubes. Serum aliquots for NRG-1 assay were stored at − 20 °C till the day of assay. NRG-1 was assayed using a human NRG-1 ELISA kit, provided by Innova Biotech Co LTD, catalog number In-Hu3368. The kit showed a sensitivity of 6 pg/ml, intra-assay precision $<$ 10% and inter-assay precision of $<$ 12%. The procedure was performed according to the manufacturer’s protocol, and results were calculated using an automated ELISA reader [44].

#### Operative phase (II)

Laparoscopic Sleeve Gastrectomy (LSG) was performed on all patients as previously described. In LSG, the esophageal fat pad was routinely removed. The first firing of the linear stapler was started, 2 cm to 4 cm from the pylorus. Stapling commenced over 40 French bougies. The first reload was a green load (3.8 mm) followed by multiple blue loads (3.5 mm) up to the Angle of His. No buttress material was used. The staple line was routinely covered with a running 3–0 Prolene (Ethicon, Inc.) suture. The specimen of the stomach was then removed. Bougie size used was of 40 FFR [45].

#### Post-operative phase (III)

After 1 month of the operation, subjects were re-questioned about their measurements; weight, height, NRG-1. (q.1–3) as well as the depression symptoms using the Arabic Version of the Beck Depression Inventory (BDI-II) [38, 39] (q 4–24).

### Statistical analysis

Collected data were statistically analyzed in IBM SPSS Statistics version 26, using the appropriate techniques to achieve the objectives of the study. The Kolmogorov- Smirnov was used to verify the normality of the distribution of variables. Comparisons between groups for categorical variables were assessed using the Wilcoxon Signed-Rank test, Marginal homogeneity test, and Chi-square test (Fisher or Monte Carlo). Mann-Whitney test was used to compare the two groups for not normally distributed quantitative variables. Pearson coefficient correlation between two normally distributed quantitative variables. The significance of the obtained results was judged at the 5% level (*p* ≤0.05).

## Results

As shown in Table 1 Obese and non-obese groups were matched regarding gender, age, level of education, occupation, and marital status, with no significant difference between the two groups in any of the previous items.

**Table 1:** Demographic characteristics of obese subjects scheduled for LSG (pre-operative) and non-obese subjects

**Characteristics**	**Obese (*n* = 108)**	**Non-obese (*n* = 100)**	**Test of Sig**	***P***
**Gender**				
Male	22 (20.4%)	32 (32.0%)	χ^2^ = 3.653	0.056
Female	86 (79.6%)	68 (68.0%)		
**Age**				
Mean ± SD	34.74 ± 8.56	32.72 ± 9.46	t = 1.614	0.108
Median (Min. – Max.)	34.0 (18.0–62.0)	30.0 (20.0–61.0)		
**Education**				
Illiterate/ read & write	0 (0.0%)	6 (6.0%)	χ^2^ = 4.008	^MC^*p* = 0.147
Secondary school	10 (9.2%)	8 (8.0%)		
University / post graduate studies	97 (89.8%)	86 (86.0%)		
**Occupation**				
Not working	27 (25.0%)	15 (15.0%)	χ^2^ = 3.222	0.073
Working	81 (75.0%)	85 (85.0%)		
**Marital status**				
Not married	36 (33.3%)	41 (41.0%)	χ^2^ = 5.400	^MC^*p* = 0.070
Married	67 (62.0%)	59 (59.0%)		
Divorced	5 (4.7%)	0 (0.0%)		

*SD* Standard deviation, *t* Student t-test, *χ*^*2*^ Chi square test, *MC* Monte Carlo, *p P* value for comparing between the studied obese subjects and non-obese subjects

Table 2 demonstrates that the mean Body Mass Index (BMI) has significantly decreased from 40.50 ± 5.04 to 34.45 ± 4.55 (pre- to post LSG respectively, *P* < 0.001).

**Table 2:** Characteristics of obese subjects scheduled for LSG (pre- and post-LSG) and non-obese subjects regarding height, weight and Body Mass Index (BMI)

**Characteristics**	**Obese (*n* = 108)**		**Non-obese (*n* = 100)**
	**Pre-LSG**	**Post-LSG**	
**Height (cm)**			
Mean ± SD.	167.8 ± 8.13	167.8 ± 8.13	167.6 ± 8.24
Median (Min. – Max.)	167 (150–188)	167 (150–188)	168.5 (150–183)
**Weight (Kg)**			
Mean ± SD.	114.4 ± 19.16	97.37 ± 17.09	68.61 ± 8.60
Median (Min. – Max.)	111.5 (85–170)	92 (70–155)	67 (50–88)
^**a**^**BMI (Kg/m**^**2**^**)**			
Mean ± SD.	40.50 ± 5.04	34.45 ± 4.55	24.37 ± 1.82
Median (Min. – Max.)	38.75 (30.48–52.53)	33.49 (25.1–47.02)	24.67 (19.03–27.73)
**t (p)**	26.348^*^ (< 0.001^*^)		

*SD* Standard deviation, *t* Paired t-test, *P P* value for comparing between obese subjects pre and post-LSG

^*^Statistically significant at *p* ≤ 0.05

^a^*BMI* Body Mass Index

**Fig. 2:**
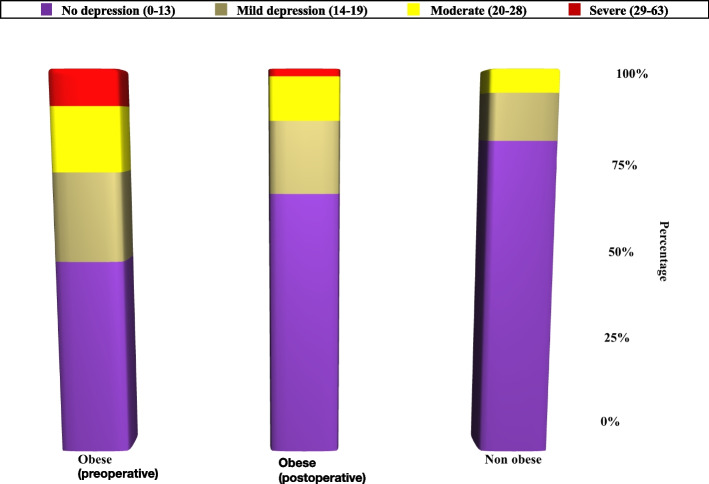
Percentage and level of depression among obese subjects (pre- and post-LSG) and non-obese subjects

**Fig. 3:**
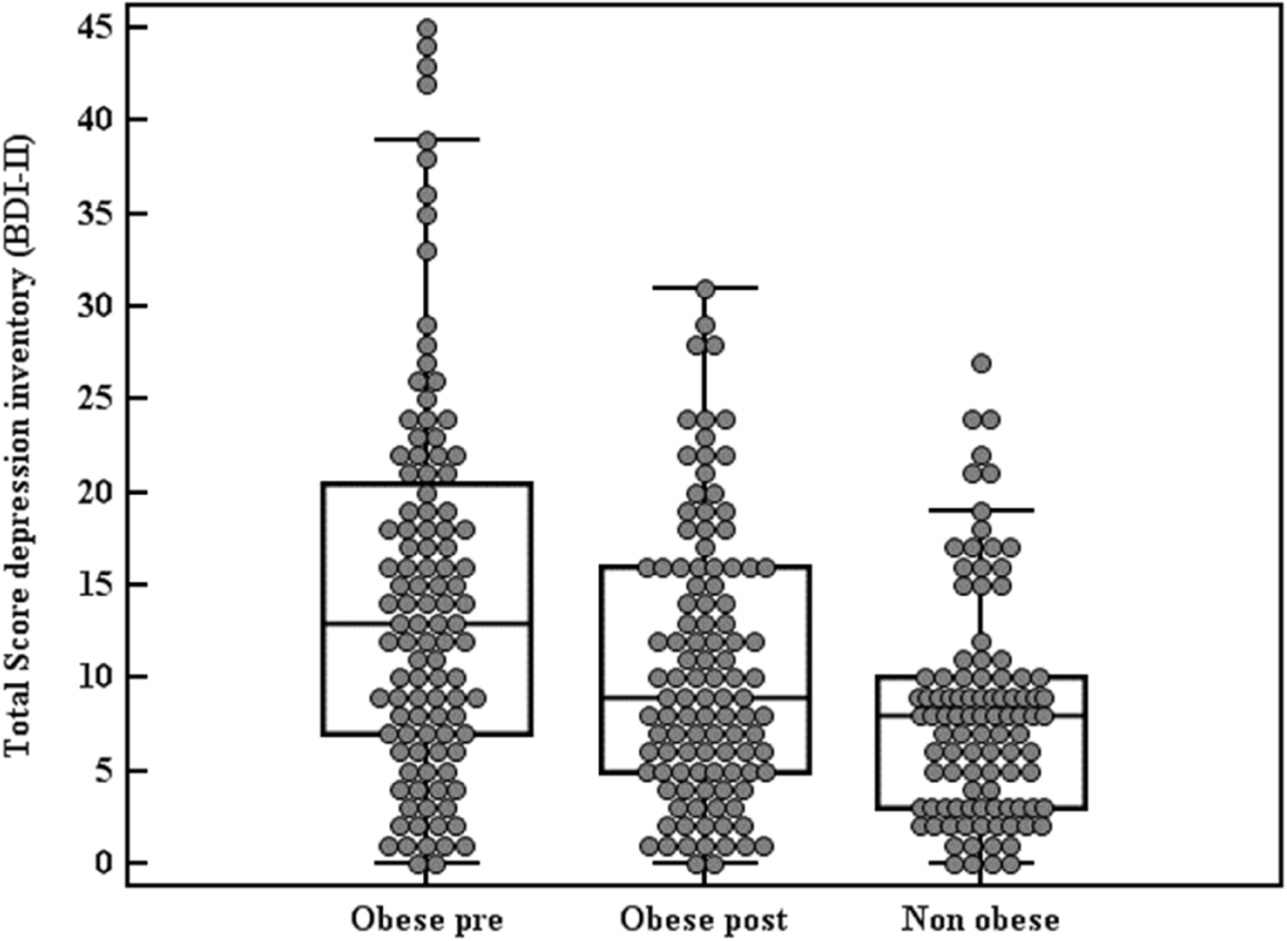
Mean values of depression symptoms of the obese subjects (pre- and post-LSG) and non-obese subjects on Beck’s depression scale (BDI-II)

Table 3, Figs. 2 and 3, represent the levels of depression in obese (percentage of depressed subjects and the total score) pre-LSG, which were significantly higher than that of the non-obese (control group), (*p* < 0.001). The severity of depression significantly decreased after the LSG. Where the depression level pre-operatively scored 9.3%, 16.7%, 23.1%, and 50.9% for severe, moderate, mild, and no depression respectively then changed post-operatively to 1.9%, 11.1%, 18.5%, and 68.5%. (*p* < 0.001).

**Table 3:** Mean values of depression symptoms of the obese subjects (pre- and post-LSG) and non-obese subjects on Beck’s depression scale (BDI-II)

**Depression Inventory (BDI-II)**	**Obese (*n* = 108)**		**Non-obese (*n* = 100)**	**Test of Sig.**	***P***
	**Pre-LSG**	**Post LSG**			
• No depression (0–13)	55 (50.9%)	74 (68.5%)	82 (82.0%)	**χ**^**2**^ = 25.619^*^	< 0.001^*^
• Mild depression (14–19)	25 (23.1%)	20 (18.5%)	12 12.0%)		
• Moderate (20–28)	18 (16.7%)	12 (11.1%)	6 (6.0%)		
• Severe (29–63)	10 (9.3%)	2 (1.9%)	0 (0.0%)		
**MH (p**_**0**_**)**	95.500^*^ (< 0.001^*^)				
**Total Score**					
Mean ± SD.	14.68 ± 10.48	10.59 ± 7.30	8.21 ± 5.98	**U** = 3326.50^*^	< 0.001^*^
Median (Min. – Max.)	13 (0–45)	9 (0–31)	8 (0–27)		
**Z (p**_**0**_**)**	5.442^*^ (< 0.001^*^)				

Test *χ*^*2*^ Chi square test, *MH* Marginal Homogeneity, *P*_*0*_*P* value for comparing between obese subjects pre- and post-LSG, *SD* Standard deviation, *U* Mann Whitney test, *Z* Wilcoxon signed ranks test

^*^Statistically significant at *p* ≤ 0.05

As illustrated in Table 4, there is a significant improvement in the depression level after LSG in the majority of the obese subjects. The pre-operative severely depressed obese subjects were 9.3% of the total obese subjects, while post-operatively, 1.9% of this percentage reported no depression and 5.6% reported moderate depression. In addition, 16.7% of the total obese subjects who suffered from moderate depression pre-operatively, improved to 5.9% with no depression and 7.4% only mildly depressed. Meanwhile, out of the 23.1% of the total obese subjects who suffered from mild depression pre-operatively, 15.7% turned out to be non-depressed post-operative. Whereas, a small percentage of the obese depressed subjects remained at the same level of depression (1.9%). Nevertheless, another percentage (5.5% of the total number) turned depressed postoperatively while they suffered no depression preoperatively.

**Table 4:** The improvement of depression level among obese subjects between pre- to post-LSG regarding Beck’s depression scale (BDI-II)

**Depression Inventory (BDI-II) (post)**	**Depression Inventory (BDI-II) (pre) (*n* = 108)**^**a**^				**χ**^**2**^	^**MC**^***P***
	**Non (*n* = 55)** **50.9%**	**Mild (*n* = 25)** **23.1%**	**Moderate** **(*n* = 18)** **16.7%**	**Severe (*n* = 10)** **9.3%**		
• Non-depressed (*n* = 74)	49 (45.4%)	17 (15.7%)	6 (5.6%)	2 (1.9%)	49.444^*^	< 0.001^*^
• Mild (*n* = 20)	5 (4.6%)	7 (6.5%)	8 (7.4%)	0 (0.0%)		
• Moderate (*n* = 12)	1 (0.9%)	1 (0.9%)	4 (3.7%)	6 (5.6%)		
• Severe (*n* = 2)	0 (0.0%)	0 (0.0%)	0 (0.0%)	2 (1.9%)		

*χ*^*2*^ Chi square test, *MC* Monte Carlo, *p P* value for association between different categories

^*^Statistically significant at *p* ≤ 0.05

^a^Percentage is calculated from the total *n* = 108

**Fig. 4:**
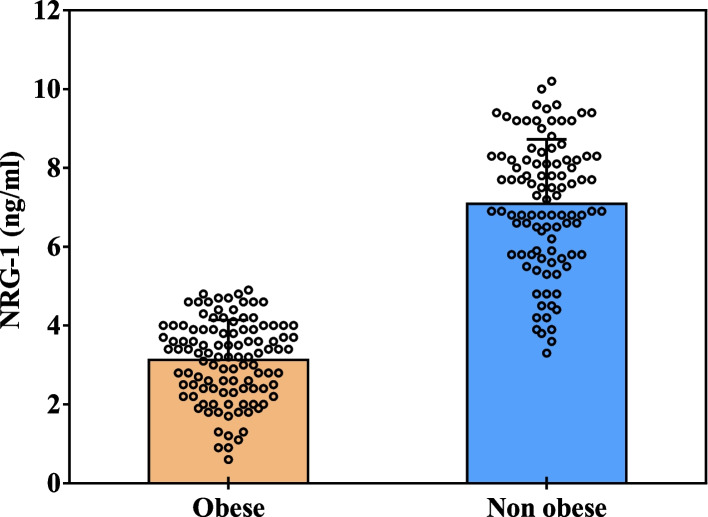
Comparison between the obese subjects (pre-LSG) and non-obese subjects regarding serum levels of NRG-1 (ng/ml)

**Fig. 5:**
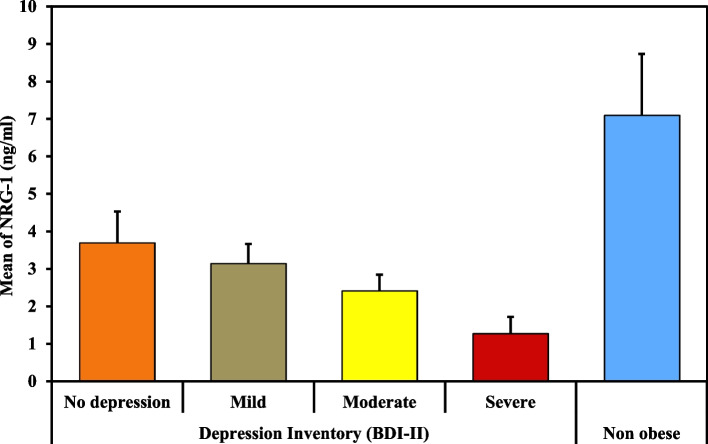
Comparison of serum NRG-1 (ng/ml) among obese subjects (pre-LSG) with different depression levels and non-obese subjects

Table 5 and Fig. 4 show that serum neuregulin 1 (NRG-1) level was significantly lower in obese persons compared to the controls. In addition, as represented in Table 6 and Fig. 5, the serum level of NRG-1 was significantly lower in severely depressed obese subjects compared to the non-obese, non-depressed obese, as well as mild and moderately depressed obese (*p* < 0.001).

**Table 5:** Comparison of the obese subjects (pre-LSG) and non-obese subjects regarding serum levels of NRG-1 (ng/ml)

**NRG-1 (ng/ml)**	**Obese (*n* = 108)**	**Non-obese (*n* = 100)**	**t**	***P***
Mean ± SD.	3.13 ± 1.01	7.09 ± 1.64	20.766^*^	< 0.001^*^
Median (Min. – Max.)	3.30 (0.60–4.90)	7.25 (3.30–10.20)		

*SD* Standard deviation, *t* Student t-test, *p P* value for comparing between obese (pre-LSG) and non-obese subjects

^*^Statistically significant at *p* ≤ 0.05

**Table 6:** Comparison of serum NRG-1 (ng/ml) among obese subjects (pre- and post-LSG) with different depression levels and non-obese subjects

**NRG-1 (ng/ml)**	**Obese (*n* = 108)**				**Non-obese (*n* = 100)**	***F***	***P***
	**Depression Inventory (BDI-II) (pre)**						
	**No depression (*n* = 55)**	**Mild depression (*n* = 25)**	**Moderate depression (*n* = 18)**	**Severe depression (*n* = 10)**			
**Mean ± SD.**	3.69 ± 0.84	3.15 ± 0.51	2.42 ± 0.43	1.28 ± 0.44	7.09 ± 1.64	140.87^*^	< 0.001^*^
**Median (Min. – Max.)**	3.90 (1.80–4.90)	3.20 (2.20–4.40)	2.40 (1.80–3.40)	1.25 (0.60–2.00)	7.25 (3.30–10.20)		

*F* F for ANOVA test, *p* *P* value for association between different levels of depression in obese and non-obese subjects

^*^Statistically significant at *p* ≤ 0.05

**Fig. 6:**
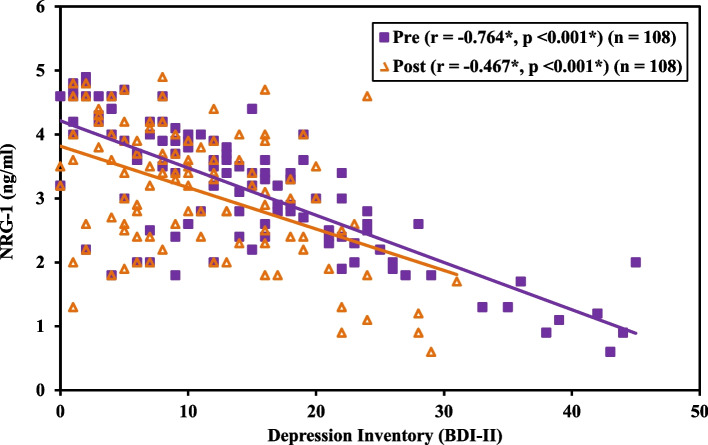
Correlation between NRG-1 (ng/ml) and Depression Inventory (BDI-II) in Obese

Table 7 and Fig. 6 illustrate a negative correlation between the serum level of NRG1 and the level of depression in obese subjects pre- and post-LSG (*r* = -0.764 and -0.467 respectively and *p* < 0.001).

**Table 7:** Correlation between NRG-1 (ng/ml) and Depression Inventory (BDI-II) in Obese (*n* = 108)

**Depression Inventory (BDI-II)**	**NRG-1 (ng/ml)**	
	***r***	***p***
**Pre**	-0.764^*^	< 0.001^*^
**Post**	-0.467^*^	< 0.001^*^

*r* Pearson coefficient

^*^Statistically significant at *p* ≤ 0.05

**Fig. 7:**
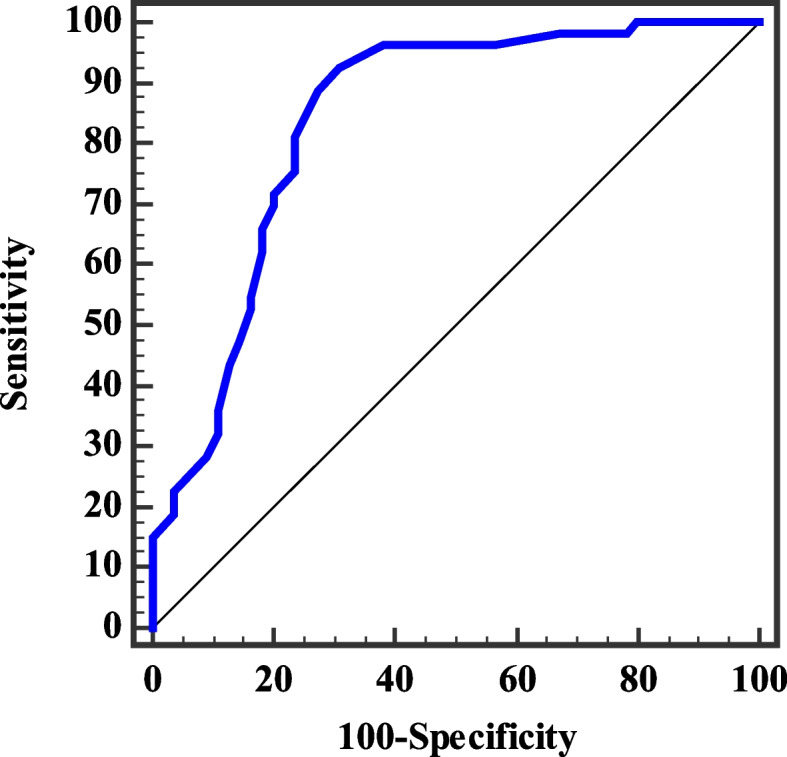
ROC curve for NRG-1 (ng/ml) as a predictor of (mild /moderate /severe) depression (*n* = 53) or no depression (*n* = 55) in pre-LSG obese subjects

**Fig. 8:**
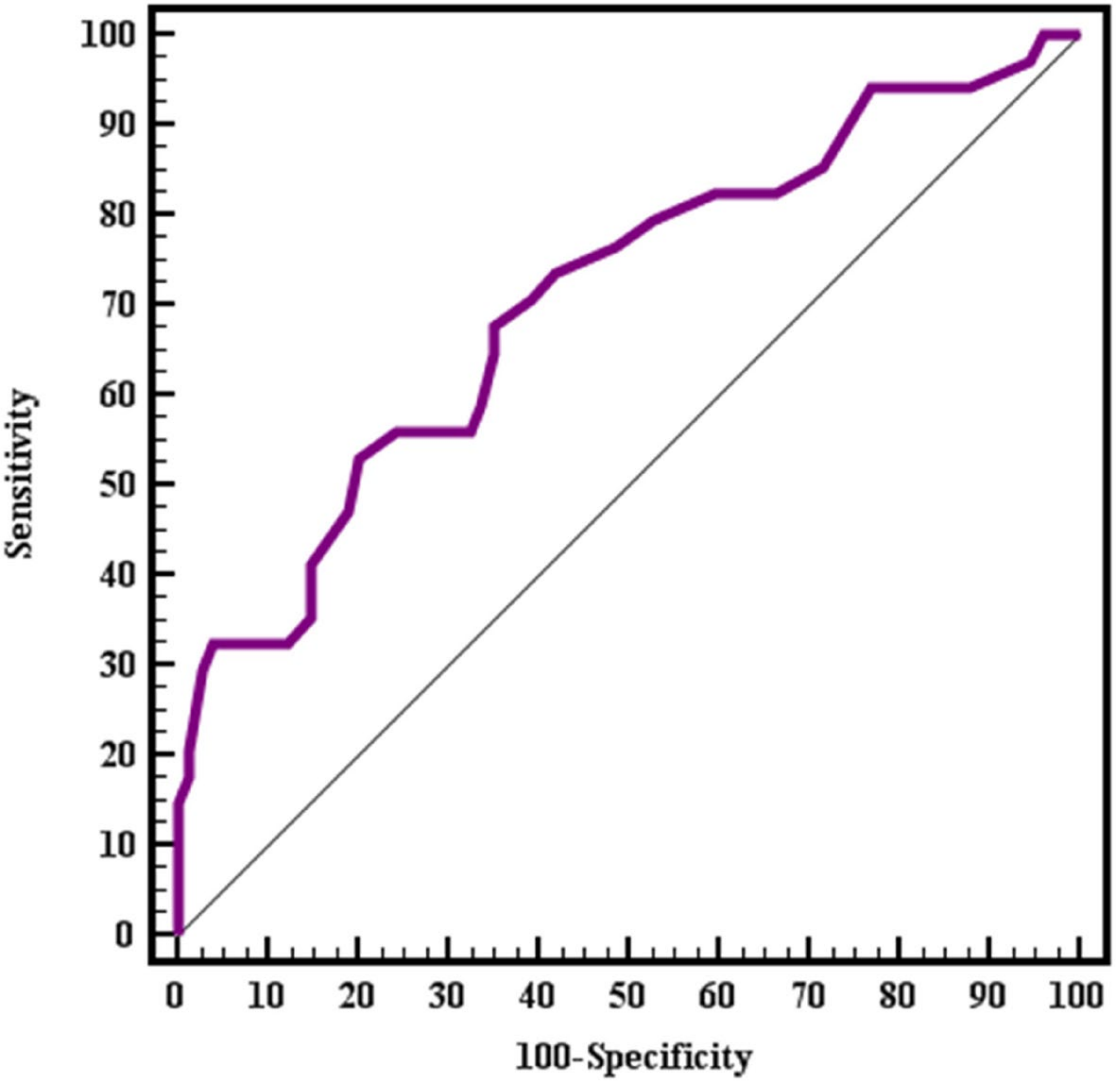
ROC curve for NRG-1 (ng/ml) as a predictor of (mild /moderate /severe) depression (*n* = 34) or no depression (*n* = 74) in post-LSG obese subjects

The sensitivity and specificity of serum NRG1 for the prediction of depression in obese persons have been determined by plotting a receiver-operating characteristic (ROC) curve (Tables 8 and 9 and Fig. 7 and 8).

**Table 8:** Validity (AUC, sensitivity, specificity) for NRG-1 (ng/ml) to discriminate between pre-LSG obese subjects with (mild /moderate /severe) depression (*n* = 53) and non-depressed subjects (*n* = 55)

	**AUC**	***P***	**95% C.I**	**Cut off**^**a**^	**Sensitivity**	**Specificity**	**PPV**	**NPV**
**NRG-1 (ng/ml)**	0.838	< 0.001^*^	0.760–0.916	≤ 3.5	92.45	69.09	74.2	90.5

*AUC* Area Under a Curve, *p value* Probability value, *CI* Confidence Intervals, *NPV* Negative predictive value, *PPV* Positive predictive value

^*^Statistically significant at *p* ≤ 0.05

^a^Cut off was choose according to Youden index

**Table 9:** Validity (AUC, sensitivity, specificity) for NRG-1 (ng/ml) to discriminate between post-LSG obese subjects with (mild /moderate /severe) depression (n = 34) and non-depressed subjects (n = 74)

	**AUC**	***P***	**95% C.I**	**Cut off**^**a**^	**Sensitivity**	**Specificity**	**PPV**	**NPV**
**NRG-1 (ng/ml)**	0.707	0.001^*^	0.598–0.816	≤ 2.5	52.94	79.73	54.5	78.7

*AUC* Area Under a Curve, *p value* Probability value, *CI* Confidence Intervals, *NPV* Negative predictive value, *PPV* Positive predictive value

^*^Statistically significant at *p* ≤ 0.05

^a^Cut off was choose according to Youden index

The sensitivity of serum NRG1 as a predictor for different levels of depression pre- and post-LSG has been estimated to be 92.45% and 52.94% respectively while its is 69.09% and 79.73% respectively at cut-off values of ≤3.5 and ≤2.5 ng/ml. These results conclude that NRG-1 has higher diagnostic and predictive efficiency for depression both pre- or postoperative (area under the curve (AUC) is 0.838 and 0.707 respectively).

The multivariate analysis in Table 10, shows that lower NRG-1 was a significant independent predictor of obesity (OR=0.073 [0.029 – 0.184], *p* = 0.001). Whereas gender was not a significant confounding factor (OR = 3.027 [0.658 – 13.926], *p* = 0.155).

**Table 10:** Multivariate logistic regression analysis for the parameters affecting obesity

	^**#**^**Multivariate**	
	***p***	**OR (LL – UL 95%C.I)**
**Gender**	0.155	3.027 (0.658–13.926)
**NRG-1 (ng/ml)**	< 0.001^*^	0.073 (0.029–0.184)

*OR* Odds ratio, *C.I* Confidence interval, *LL* Lower limit, *UL* Upper Limit

^#^All variables with *p* < 0.05 was included in the multivariate

^*^Statistically significant at *p* ≤ 0.05

## Discussion

Obesity and depression both impact health tremendously. Data is implying that depression could be a risk factor for obesity, as depressed patients taking a high-fat diet have higher rates of obesity than non-depressed people on the same diet. Vice versa, obesity may increase the risk of depression [46] as people suffering from obesity somehow have body image concerns and stigmatization [14]. The association between depression and obesity could be attributed to certain genes involved in both pathologies [46]. Afari et al. found that 12% of the genetic component of depression is shared by obese persons [47].

Our analysis has demonstrated that depression was significantly higher among obese compared to non-obese patients in the preoperative period. After the operation, 1.9% of the severely depressed subjects reported no depression, while 5.6% became moderately depressed; about 6% of the moderately depressed and 16% of the mildly depressed became not depressed. Serum NRG-1 level was significantly lower among obese and severely depressed compared to the controls.

In the current study, we aimed to investigate the role of neuregulin-1 as a potential blood biomarker linking depression to obesity among obese subjects assigned for laparoscopic sleeve gastrectomy. We found that depression symptoms decreased significantly in obese subjects 1 month after undergoing LSG. Moreover, there was a significantly higher score of depression among obese subjects compared to controls with normal weight. In addition, a high total score of depression was associated with high BMI in obese persons pre- and post-LSG. That was consistent with Amiri & Behnezhad who found that depressive symptoms were significantly more frequent in obese and overweight people than those having normal body weight [2]. As well that matches the results of Luppino et al. and de Wit et al. in their meta-analysis of community-based cross-sectional studies [48, 49].

Our results revealed a decrease in BMI 1 month after the LSG. The reduction of weight was accompanied by a significant reduction of depression symptoms in most of the study subjects. This improvement might not be directly due to the resulting weight loss, but the surgery may somewhat trigger behavioral, physiological, and cognitive changes involving the improvement of body image that generally raises both physical and psychosocial outcomes [50]. Weight loss might also play a role to reduce psychological distress, deactivate inflammatory pathways and correct the function of the hypothalamic-pituitary axis (HPA) axis [51].

We detected a small percentage in our study that showed no improvement or even an increase in depression symptoms post-LSG. In the same context Angrisan et al. and Jumbe et al. reported that there is relative individual variation in weight reduction after surgery, and some patients might experience worsening of their psychological health status [52, 53].

This could be explained by the lack of support [54] or pain perceived by some subjects interfering with synaptic connectivity at the prefrontal cortex [55] and hippocampus [56], altering dopamine and serotonin (5HT) signaling [57, 58].

Another explanation could be the concern of loss of self-identity after surgery and not being identified by their surrounding people [59]. Nevertheless, over-expectation of excessive weight loss postoperatively could increase depression symptoms if not met as imagined [51].

So far, according to available information, our study has dug to somehow provide detailed insight into the mechanisms linking depression and obesity, where it spotted a significant decrease of serum NRG-1 level in obese people compared to non-obese, at the same time its levels correlated negatively with the score of depression in obese both pre- and post-LSG. These results agreed with the findings of the experimental studies performed on depression previously [23, 30–32].

As NRG-1 is distributed in the frontal cortex, cerebellum, and midbrain, and has a role in synaptic plasticity [30]. Low NRG-1 in the cortical projection neurons contributes to increased inhibitory connections and lower synaptic plasticity [31, 32], eventually increasing the individual’s susceptibility to stress-induced depression and affecting the emotional response in general [23].

Regarding our findings of the association of reduced NRG-1 with obesity, Wang et al. [59] reported that NRG-1 treatment experimentally, improved glucose tolerance in diabetic mice [60], which further supports enhancing NRG-1 pathway as a promising treatment for insulin resistance besides its role in oxidative metabolism [61, 62].

Moreover, the NRG‑1/ErbB pathway enhances leptin levels, enlightening the possible approach of exploring underlying mechanisms of action of NRG‑1 in a myocardial IR model with obesity or a high‑fat diet [61–63].

## Conclusion

Due to its multiple functions in health and disease, NRG-1 could be further studied as a marker or in treatment [64]. From the observation of our study we suggest that NRG-1 is a possible biomarker for the diagnosis of depression pre-bariatric surgery and the prediction of its prognosis post-operatively. Further studies are needed to investigate the relationship between depressive symptoms and NRG-1 levels 3 and 6 months postoperatively.

## Data Availability

Data is available upon reasonable request to the corresponding author.

## Abbreviations

WHO: World health organization
NRG: Neuregulins
NRG-1: Neuregulin-1
EGF: Epidermal growth factor
CNS: Central nervous system
LSG: Laparoscopic sleeve gastrectomy
BMI: Body mass index
ELISA: Enzyme-linked immunosorbent assay
BDI-II: Beck Depression Inventory-II
DSM-IV: Diagnostic and statistical manual of mental disorder – 4^th^ edition
ROC: Receiver-operating characteristic
AUC: Area under curve
HPA: Hypothalamic pituitary axis
5HT: Hydroxytryptamine
Ng/ml: Nanogram per milliliter
